# Titanium dioxide nanoparticles strongly impact soil microbial function by affecting archaeal nitrifiers

**DOI:** 10.1038/srep33643

**Published:** 2016-09-23

**Authors:** Marie Simonin, Agnès Richaume, Julien P. Guyonnet, Audrey Dubost, Jean M. F. Martins, Thomas Pommier

**Affiliations:** 1 Univ Lyon, Université Claude Bernard Lyon 1, UMR CNRS 5557, Laboratoire d’Ecologie Microbienne, UMR INRA 1418, bât G. Mendel, 43 boulevard du 11 novembre 1918, F-69622 Villeurbanne Cedex, France; 2 LTHE, UMR 5564 CNRS – Univ. Grenoble Alpes 38041 Grenoble Cedex 9, France

## Abstract

Soils are facing new environmental stressors, such as titanium dioxide nanoparticles (TiO_2_-NPs). While these emerging pollutants are increasingly released into most ecosystems, including agricultural fields, their potential impacts on soil and its function remain to be investigated. Here we report the response of the microbial community of an agricultural soil exposed over 90 days to TiO_2_-NPs (1 and 500 mg kg^−1^ dry soil). To assess their impact on soil function, we focused on the nitrogen cycle and measured nitrification and denitrification enzymatic activities and by quantifying specific representative genes (*amoA* for ammonia-oxidizers, *nirK* and *nirS* for denitrifiers). Additionally, diversity shifts were examined in bacteria, archaea, and the ammonia-oxidizing clades of each domain. With strong negative impacts on nitrification enzyme activities and the abundances of ammonia-oxidizing microorganism, TiO_2_-NPs triggered cascading negative effects on denitrification enzyme activity and a deep modification of the bacterial community structure after just 90 days of exposure to even the lowest, realistic concentration of NPs. These results appeal further research to assess how these emerging pollutants modify the soil health and broader ecosystem function.

Titanium dioxide nanoparticles (TiO_2_-NPs) are widely used in commercial products such as sunscreens and toothpastes, industrial products like paints, lacquers and paper, and in photocatalytic processes such as water treatment^1^,^2^,^3^,^4^. Consequently, TiO_2_-NPs are indirectly discharged in agricultural soils through irrigation or sewage-sludge application^5^ and directly as nanofertilizers or nanopesticides^6^. Despite their importance in soil ecosystem function, the current literature lacks thorough investigations of the effect of TiO_2_-NPs on soil microbial communities^7^.

Microbial communities play key roles in plant productivity and in biogeochemical processes^8^,^9^,^10^,^11^, such as the nitrogen (N) cycle, in which nitrification and denitrification processes control soil inorganic N availability and subsequent soil fertility^10^. The strong coupling between nitrification and denitrification makes the N cycle an ideal model to study the impacts of environmental disturbances on microbial functioning. Despite the recent descriptions of complete single-organism oxidation from ammonium (NH_4_^+^) to nitrate (NO_3_^−^) in some members of the *Nitrospira* genus^12^,^13^, nitrification is usually considered a two-step aerobic process. The current paradigm is that the rate-limiting step in nitrification is the oxidation of NH_4_^+^ into nitrite (NO_2_^−^)^14^, performed by both ammonia-oxidizing bacteria (AOB) and ammonia-oxidizing archaea (AOA)^15^. Nitrification is carried out by a group of microorganisms exhibiting a low phylogenetic diversity and is one of the most sensitive soil microbial processes to environmental perturbations, such as pollutant exposure^16^,^17^. In contrast to the impacts on AOB function, the influence of environmental stressors on AOA is less known^18^,^19^ as they were only recently identified as pivotal in soil nitrification^20^.

Under anaerobic conditions, nitrite and/or nitrate can be sequentially reduced into gaseous products (NO, N_2_O and N_2_)^21^ during the denitrification process. Contrarily to nitrification, the ability to perform denitrification is widespread among several phylogenetic groups and this functional guild has been shown to be moderately insensitive to some toxicants^22^. However, the sensitivity of denitrifiers to TiO_2_-NPs has not been investigated. Moreover, despite a potential resistance of the denitrifiers to this pollutant, a failure of nitrification may still impose scaffolding impacts resulting in decreased denitrification.

Some studies observed decreased overall microbial respiration and enzyme activity in soils exposed to high TiO_2_-NPs concentrations^23^,^24^,^25^. However, the targeted impact of TiO_2_-NPs on the N cycle, regulated by microbial communities of varying degrees of functional redundancy, has not been investigated in soils.

Here we report the effects of TiO_2_-NPs on an agricultural soil (silty-clay texture) subjected to a 90 d exposure of two TiO_2_-NPs concentrations, simulating either an environmentally realistic contamination (1 mg kg^−1^ dry soil) or an accidental pollution (500 mg kg^−1^ dry soil)^26^. A previous study demonstrated that among six soils of varying degrees of texture and organic matter content, silty-clay soil presented the highest effects of TiO_2_-NPs on soil respiration due to the lower stability of NP aggregates in the soil solution^25^. To further investigate the impact of TiO_2_-NPs on soil function, we examined their effects on nitrification and denitrification enzymatic activities (NEA and DEA, respectively), along with the quantification of the representative genes of the functional guilds performing these activities by quantitative PCR (*i.e. amo*A for ammonia-oxidizers and *nirK* and *nirS* for denitrifiers). Additionally, the effects of TiO_2_-NPs on the microbial diversity was determined by targeting the 16S rDNA bacterial and archaeal genes and *amo*A AOA and AOB genes with high throughput sequencing (MiSeq, Illumina). We developed a path analysis to integrate the different variables measured in our experiment to assess the multifaceted consequences of TiO_2_-NPs on the N cycle in an agricultural soil.

## Results

### Impact of TiO_2_-NPs on soil function

NEA and DEA were monitored after 0, 7, 30 and 90 d of incubation. As expected after 90 d in a microcosm experiment, NEA and DEA decreased over time in the controls (−17% and −16%, respectively, Fig. S1). The impacts of TiO_2_-NPs exposures were therefore considered as percentage relative to the controls.

**Figure 1:**
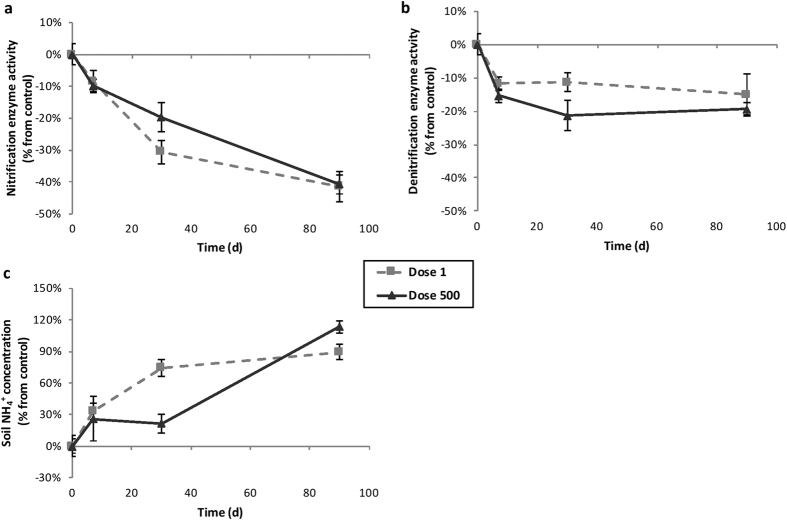
Changes over time of NEA (**a**), DEA (**b**,**c**) soil NH_4_^+^ concentration in the different treatments (dash line: Dose 1 mg kg^−1^, black line: Dose 500 mg kg^−1^ dry soil). Results are expressed as percentage relative to the controls and error bars represent the standard error (n = 6).

After 30 d of exposure, both TiO_2_-NPs concentrations reduced NEA compared to the control with 31% decrease (*P* < 0.001) and 20% (*P* = 0.02) for 1 and 500 mg kg^−1^, respectively (Fig. 1a). These reductions were more pronounced after 90 days with about 40% decrease (*P* < 0.001) for both concentrations. The inhibiting effect of TiO_2_-NPs on NEA triggered a significant accumulation of NH_4_^+^ after 30 d for 1 mg kg^−1^ (Fig. 1c, +74%, *P* = 0.01) and for both concentrations after 90 d (1 mg kg^−1^: +89%, *P* < 0.001; 500 mg kg^−1^: +113%, *P* = 0.001). Moreover, soil NH_4_^+^ concentration was negatively correlated to NEA after 30 (*r* = 0.57, *P* = 0.01) and 90 d (*r* = 0.83, *P* < 0.001).

DEA significantly decreased on the 3 sampling dates with 500 mg TiO_2_-NPs kg^−1^ (Fig. 1b, 7 d: −15%, *P* = 0.01; 30 d: −21%, *P* = 0.04; 90 d: −19%, *P* < 0.001). The soil exposure with 1 mg kg^−1^ resulted in a significant reduction of DEA only after 90 d of incubation (−15%, *P* < 0.001).

### Impact of TiO_2_-NPs on N-related functional guild abundances

**Figure 2:**
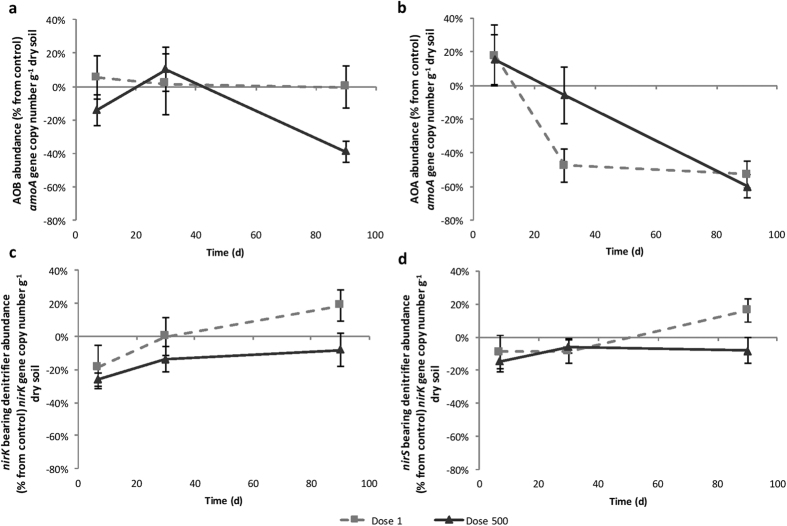
Changes over time of (**a**) ammonia-oxidizing bacteria (AOB), (**b**) ammonia-oxidizing archaea (AOA), (**c**) *nirK* bearing denitrifier abundance and (**d**) *nirS* bearing denitrifier abundance in the different treatments (dash line: Dose 1 mg kg^−1^, black line: Dose 500 mg kg^−1^ dry soil). Results are expressed as percentage relative to the controls and error bars represent the standard error (n = 6).

Overall, the abundance of *amoA* AOA gene was 75 fold higher than corresponding *amoA* AOB abundance (average AOA and AOB: 9.27 × 10^7^ and 1.25 × 10^6^ copies g^−1^ dry soil, respectively). The dynamics of *amoA* AOA and AOB under TiO_2_-NP treatments were contrasted (Fig. 2): while the soil exposure at 500 mg kg^−1^ TiO_2_-NPs lead to a decrease of *amoA* AOB abundance only after 90 d (Fig. 2a, −39%, *P* = 0.05), no variation was observed at 1 mg kg^−1^ during the course of the experiment. On the contrary, the abundance of *amoA* AOA strongly decreased regardless of the applied concentration after 90 d (Fig. 2b, 1 mg kg^−1^: −53%, *P* < 0.001; 500 mg kg^−1^: −60%, *P* < 0.001). NEA was negatively correlated with the abundance of *amoA* AOB (*r* = 0.42, *P* = 0.002, Table 1). In contrast, NEA was positively correlated with the abundance of *amoA* AOA (*r* = 0.51, *P* < 0.001, Table 1). In addition, soil NH_4_^+^ concentration was strongly negatively correlated with *amoA* AOA abundance after 90 d (*r* = 0.88, *P* < 0.001).

The abundance of *nirS* gene was 4.5 fold higher than the abundance of *nirK*, with an average of 1.15 × 10^7^ and 2.57 × 10^6^ copies g^−1^ dry soil, respectively. The *nirK* and *nirS* gene abundances were not significantly impacted by TiO_2_-NPs exposure (Fig. 2c,d). DEA was not correlated to the abundance of *nirS* but was positively correlated with the abundance of *nirK* (R^2^ = 0.28, *P* < 0.001, Table 1).

### Impact of TiO_2_-NPs on soil microbial diversity

**Figure 3:**
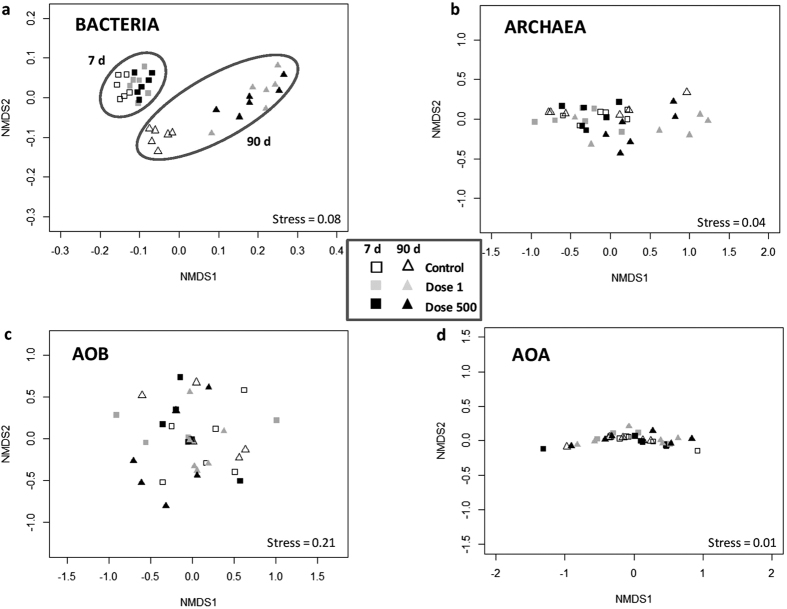
Nonmetric Multidimensional Scaling (NMDS) analysis to determine the modification in microbial community structure in presence of TiO_2_-NPs (white: Control, grey: Dose 1, black: Dose 500) after 7 d (square symbols) and 90 d (triangle symbols): (**a**) bacterial community, (**b**) archaeal community, (**c**) AOB community, (**d**) AOA community.

We assessed the impact of TiO_2_-NPs on soil microbial diversity by sequencing the 16S rDNA and *amoA* bacterial and archaeal genes (Table 2). The sequencing effort results in 160,301 16S rDNA bacterial sequences comprising 8,256 Operational Taxonomic Units (OTUs) (per soil sample: 1,466 ± 8.85 OTUs) at a distance dissimilarity of 0.03. No significant change in 16S rDNA richness (ACE estimator) was observed among the soil samples. However, the TiO_2_-NPs exposure dosage and the time of exposure, as well as the interaction between these parameters, caused a significant shift in the bacterial community structure (TiO_2_-NPs: *P* < 0.001, time: *P* < 0.001, TiO_2_-NPs × time: *P* = 0.005, Fig. 3a). Both doses of TiO_2_-NPs significantly modified the bacterial community structure (1 mg kg^−1^: *P* = 0.001, 500 mg kg^−1^: *P* = 0.003) after 90 d and the relative abundance of several OTUs was either reduced (e.g. *Acidobacteria* group 6, *Saprospirae*) or increased (e.g. *Sulfuritalea*, *Anaerolinea*, *Bacteroidia*) compared to the control (Fig. S2). After 90 d, the shift observed in the bacterial community structure was associated with a modification of the relative abundance of different phyla, in particular the *Proteobacteria*, *Acidobacteria*, *Actinobacteria* and *Chloroflexi* phyla (Fig. S3).

**Figure 4:**
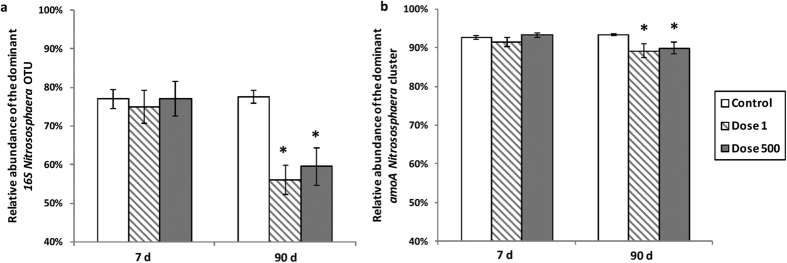
Effects of TiO_2_-NPs on the relative abundance (**a**) of the dominant archaeal 16S rDNA OTU affiliated to the *Nitrososphaera* genus, (**b**) of the dominant AOA *amoA* cluster affiliated to the *Nitrososphaera* cluster. The mean and standard errors are presented (n = 6) and the treatments are represented as: white bars: Control, hashed bars: Dose 1 mg kg^−1^, dark grey bars: Dose 500 mg kg^−1^ dry soil.

The archaeal community diversity, also inferred from the 16S rDNA gene, had low richness with a total of 364 OTUs (per soil sample: 28.75 ± 0.91 OTUs) from 206,831 archaeal sequences delineated at a dissimilarity distance of 0.03. The archaeal communities were universally dominated by a single OTU, with a relative abundance of 70.4 ± 2%. This ubiquitous OTU belongs to an ammonia-oxidizing archeon of the genus *Nitrososphaera* (Fig. S4). The archaeal community structure was modified over time (*P* = 0.002, Fig. 3b), but no community changes were detected with TiO_2_-NP exposure treatments (*P* = 0.34). The relative abundance of the dominant *Nitrososphaera* OTU was significantly reduced in the microcosms exposed to both concentrations of TiO_2_-NPs after 90 d (1 mg kg^−1^: −28%, *P* < 0.001; 500 mg kg^−1^: −23%, *P* < 0.001, Fig. 4a). Interestingly, the relative abundance of this OTU was positively correlated to the abundance of *amoA* AOA (*r* = 0.89, *P* < 0.001) and to NEA (*r* = 0.62, *P* = 0.02). After 90 d under TiO_2_-NP exposure, while the relative abundance of the dominant *Nitrososphaera* OTU decreased, the relative abundance of a *Methanocella* OTU increased by >11.5% (from 0.3 ± 0.08% in the control to 12 ± 3%) as a result of TiO_2_-NPs exposure (Fig. S4).

The 422,844 *amoA* AOB gene sequences clustered into 5974 total observed clusters (per soil sample: 272 ± 2.5 clusters) at a dissimilarity of 0.05. The ACE richness estimator of the AOB community significantly decreased after 90 d in presence of 500 mg kg^−1^ of TiO_2_-NPs (−8.4%, *P* = 0.004). However, the AOB community structure did not vary among TiO_2_-NP treatments throughout the incubation (TiO_2_-NPs: *P* = 0.68, time: *P* = 0.21, Fig. 3c).

The 609,456 *amoA* AOA gene sequences yielded 2,103 total clusters (per soil sample: 44 ± 8 clusters) at a dissimilarity of 0.05. The AOA community structure assessed by *amoA* AOA gene sequences was neither modified when exposed to TiO_2_-NPs nor during the incubation (TiO_2_-NPs: *P* = 0.95, time: *P* = 0.31, Fig. 3d). As observed for the 16S rDNA, a single cluster belonging to *Nitrososphaera* cluster dominated the *amoA* AOA sequences in all samples, representing on average 91% of all *amoA* AOA sequences analyzed (Fig. S5). The other clusters obtained were also in the genus *Nitrososphaera* (Fig. S5). The relative abundance of the dominant *amoA Nitrososphaera* cluster was slightly decreased by TiO_2_-NP exposure after 90 d (Fig. 4b, −4%, *P* < 0.001 for both concentrations).

### Path Analysis: Integrating the relationships among TiO_2_-NP exposure, microbial communities and soil function

**Figure 5:**
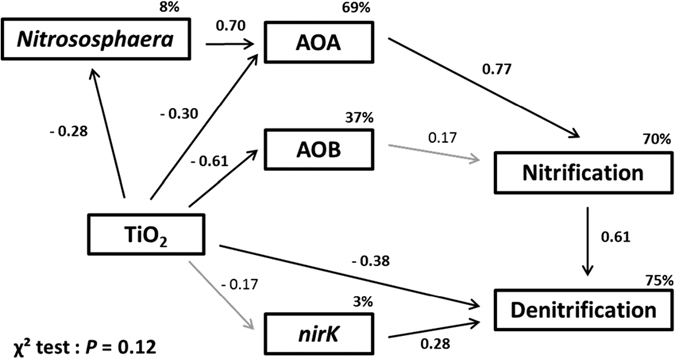
Path analysis of the direct and indirect effects of TiO_2_-NPs, dominant 16S rDNA *Nitrososphaera* sequence number, AOA abundance, AOB abundance, *nirK* abundance on NEA and DEA. Path coefficients (values adjacent to the arrows) correspond to the standardized coefficients calculated based on the analysis of correlation matrices and indicate by how many standard deviations the effect variable would change if the causal variable was changed by one standard deviation. Arrows and values in bold indicate a significant causal relationship between two variables.

We investigated possible causal relationships between TiO_2_-NPs exposure and microbial activity, abundance and diversity using a path analysis (Fig. 5). The path analysis was performed on the data set obtained after 90 d of exposure to TiO_2_-NPs since the greatest effects of TiO_2_-NPs on these soil microorganisms occurred at the 90 d sampling time (Figs 1 and 2). The variables included in the model were significantly correlated to NEA or DEA. The model explains a significant part of NEA (70%) and DEA (75%) variances. TiO_2_-NPs significantly and negatively affected the relative abundance of the dominant 16S rDNA OTU affiliated with *Nitrososphaera* (path coefficient = −0.28, *P* = 0.02) and also of *amoA* AOA and AOB overall gene abundances (path coefficient = −0.30, *P* = 0.03 and −0.61, *P* = 0.001 respectively). The dominant archaea *Nitrososphaera* was also an important driver of the AOA abundance (path coefficient = 0.70, *P* < 0.001). The path analysis supported the relative abundance of *amoA* AOA genes as the major driver of NEA in this soil (path coefficient = 0.77, *P* < 0.001) and the relative abundance of *amoA* AOB as a minor contributor to NEA (path coefficient = 0.17, *P* = 0.23). A large part of the variance of NEA was explained by indirect effects of the ubiquitous 16S rDNA *Nitrososphaera* OTU (indirect effect = 0.53). TiO_2_-NPs directly reduced DEA (path coefficient = −0.38, *P* = 0.007) but did not indirectly influence DEA through *nirK* relative abundance (indirect effects = −0.05) although *nirK* abundance was a driver of DEA (path coefficient = 0.28, *P* = 0.03). The model revealed that the DEA was mainly explained by the NEA activity (path coefficient = 0.61, *P* < 0.001). If the path between the NEA and the DEA was not included in the model, only 44% of DEA variance was explained (data not shown), emphasizing that TiO_2_-NP exposure strongly affects denitrification process through a decrease of AOA abundance and consequently NEA activity.

## Discussion

TiO_2_-NPs impose strong perturbations of the nitrogen cycle and a modification of the bacterial community structure in an agricultural soil, even at low realistic concentration (1 mg kg^−1^ dry soil). Surprisingly, the two TiO_2_-NPs concentrations used (1 and 500 mg kg^−1^ dry soil) resulted in similar effects on the soil microbial activities and AOA abundance. Non classical dose-response seems to be rather common with NPs and has been observed several times on soil microbial activities^24^,^25^,^27^. The current hypothesis is that the NPs homo- and hetero-aggregation processes (i.e. the aggregation of NPs with themselves and the aggregation of NP with other environmental constituents) vary according to NP concentration at time of exposure^28^, resulting in variable NP bioavailability and toxicity for microorganisms^29^. Moreover, the initial particle size, coating and phase composition can affect NPs reactivity and aggregation^29^,^30^. Therefore, further research on the physicochemical properties of NPs in soils as it relates to the applied concentration is necessary to clarify this assumption.

No functional resilience was observed during the time course of the experiment, which raises concerns about the ecotoxicity of TiO_2_-NPs in soils. The greatest effects of TiO_2_-NPs appeared 90 d after the exposure suggesting that aged NPs can affect microorganisms even at low concentrations and after a long exposure. This should be considered with regards to transport experiments suggesting that TiO_2_-NPs exhibit a low mobility in soils and would have a long residence time in this ecosystem^31^,^32^. Most studies to date are based on shorter incubations no longer than 60 d and simulate exposures to exceptionally high NPs concentrations (>100 mg kg^−1^)^7^. Therefore, our results demonstrate that shorter-term experiments may not accurately reflect the toxic potential of NPs in soil over the long term, suggesting that further research should be conducted under more realistic NP concentrations and assessed over longer periods.

The AOA abundance was highly reduced by TiO_2_-NP exposure compared to the AOB abundance even with the lowest concentration. In this case, the AOA abundance decreased to 60% after 90 d, presumably explaining the high accumulation of NH_4_^+^ in soil. Consistent with Mertens *et al.*^18^, our results challenge the view that archaea are more tolerant to chronic stresses than bacteria^33^,^34^. The study of AOA ecology in soil and their response to environmental stressors is still largely unexplored yet is likely an important influence on soil health^18^,^19^,^35^,^36^,^37^. The response of archaea to metal oxide NPs was studied on waste activated sludge and consistent with our study, AOAs were more affected by NPs after long-term exposure^38^,^39^. Pure culture studies have shown that TiO_2_-NPs can be toxic to bacteria after adsorption to cell membrane causing oxidative stress associated to reactive oxygen species (ROS) production^40^,^41^ and osmotic stress^42^. However, the very limited knowledge of soil archaeal physiology and the absence of targeted toxicological studies on soil archaeal strains, prevent us to say that the same toxicity mechanisms are operating on the mortality of archaea and bacteria exposed to TiO_2_-NPs.

In this study, the archaeal diversity was very low compared to bacterial diversity and the most represented soil archaea (16S rDNA) and AOA (*amoA*) were affiliated with the same one *Nitrososphaera* phylotype. This observation is consistent with the literature reporting that this AOA is dominant in many agricultural and natural soils worldwide^43^,^44^,^45^,^46^. Interestingly, this dominant 16S rDNA *Nitrososphaera* OTU decreased with TiO_2_-NPs exposure (−28 and −23%) and was positively correlated to NEA and AOA abundance. In contrast, the relative abundance of the dominant *amoA Nitrososphaera* cluster was only slightly decreased by both doses (−4%). This result demonstrates that there is a lower level of diversity for *amoA* AOA genes than for 16S rDNA genes^47^ and suggests that TiO_2_-NPs affected taxonomically distinct AOA groups harboring similar *amoA* genes. Similarly, for AOB community, we did not observe any particular pattern of *amoA* AOB community structure despite substantial shifts in the bacterial community inferred from the 16S rDNA dataset.

Based on our results, we can assume that dominant AOA *Nitrososphaera* phylotypes are the main functional drivers of nitrification in the examined soil. This is supported by the positive correlations of AOA abundance with NEA but also by the negative correlation with soil NH_4_^+^ concentration and the much higher abundance of AOA which was 75 fold greater than AOB abundance. Higher abundance of AOA than AOB in soils exhibiting neutral or alkaline pH have been reported several times^20^,^48^. Our results confirm these observations and point out that AOA can dominate ammonia-oxidizing activity under alkaline pH and high organic matter content^48^,^49^,^50^,^51^,^52^. As illustrated by the path analysis, TiO_2_-NPs had negative effects on *Nitrososphaera* relative abundance and AOA abundance with cascading negative effects on nitrification and denitrification activities. Altogether, these results suggest the pivotal role of AOA *Nitrososphaera* in the response of this soil to TiO_2_-NP contamination exposure.

Unlike the nitrifying community, the abundance of the denitrifying community was not suppressed by TiO_2_-NP exposure, yet DEA decreased (around −20%) with both TiO_2_-NP concentrations during the incubation. The higher resistance and resilience of denitrifiers to pollutants is well supported and can be explained by their high functional redundancy, niche breadth and adaptive ability^22^. The path analysis indicated that decreased denitrification was primarily explained by indirect effects through TiO_2_-NP-mediated suppression of nitrification caused by a decrease of the AOA community. However, the decline of nitrification did not fully explain the denitrification decrease since TiO_2_-NPs also directly affected denitrification (path coefficient = −0.38). This path reflects that other variables that were not included in our model are likely involved in the decrease of DEA. As suggested by Cantarel *et al.*^53^, specific clusters of denitrifying bacteria could better be related to denitrification than functional gene abundance (*nirK* or *nosZ*)^53^.

Nanomaterials such as TiO_2_-NP are worrying emerging contaminants of terrestrial ecosystem. Their effects on soil function as well as on microbial diversity and abundance of key functional guilds are of particular interest for environmental risk assessment. Our results highlight the complexity of the effects that TiO_2_-NP exposure imposes on soil microorganisms and the N cycle. Considering the functional links and indirect effects on key players of the N cycle provides an integrative assessment of their impact on the soil microbial community and its function. Particularly, soil nitrification and ammonia-oxidation performed by AOA, justify more thorough examinations from both toxicological and environmental perspectives.

## Methods

### Nanoparticles, soil and experimental design

The TiO_2_-NPs (80% anatase, 20% rutile) were provided by Sigma Aldrich (St Louis, USA) with a particle size of 21 nm in powder and ≥99.5% purity. The aggregated size and surface charge of TiO_2_-NPs in water were previously characterized by Dynamic Light Scattering (DLS) using a NanoZS (Malvern)^25^. The average aggregated size and zeta potential of TiO_2_-NPs in the ultrapure water spiking suspension were 160 ± 7.2 nm and −13.4 ± 0.5 mV, respectively.

The soil was sampled from the upper 20 cm layer of a permanent pasture at Commarin (Côte d’Or, France), sieved (2 mm) and stored at 4 °C before use. It is a silty-clay soil comprised of 39.1% clay, 50.8% loam and 10.1% sand, with an organic matter content of 7.87%, Cation Exchange Capacity (CEC) of 20.1 cmol^+^ kg^−1^ and the water content at field capacity was 51%. The soil pH (7.7) and the ionic strength (1.37 mM) were measured following ISO 10390 and ISO 11265 procedures, respectively. The pH was not modified by the addition of TiO_2_-NPs throughout the experiment (Data not shown).

The soil was exposed to one dose of 0 mg, 1 mg, or 500 mg kg^−1^ of TiO_2_-NPs suspended in ultrapure water and dispersed in an ultrasonic bath (Bioblock Scientific) for 5 min just before use. TiO_2_-NPs suspensions were added homogeneously using a multichannel pipette in order to achieve the water holding capacity. The soil was thoroughly mixed manually for 10 min to ensure a homogeneous distribution of NPs. Control soils received only ultrapure water to achieve the same final moisture. Fifty grams (equivalent dry weight) of contaminated soils were then placed into microcosms (150 ml plasma flask) and sealed with rubber stoppers. Soil microcosms were incubated for 7, 30 or 90 days at 28 °C in the dark and were weekly aerated to ensure a renewal of the atmosphere in the flasks. The experimental design consisted of 54 microcosms: 3 concentrations of TiO_2_-NPs (0, 1, 500 mg kg^−1^ dry soil) ×3 exposure times ×6 replicates per treatment. At the beginning of the experiment, six additional microcosms were prepared to assess the baseline conditions of the soil. For each incubation time, the microcosms were destructively harvested and soil subsamples were immediately assessed for NEA and DEA measurements, and 3 g were stored at −20 °C until DNA extraction.

### Nitrification Enzymatic Activity (NEA) and soil NH_4_^+^ concentration

NEA was determined following the protocol described in Dassonville *et al.*^54^. Subsamples of fresh soil (3 g dry soil) were incubated with 6 ml of a solution of (NH_4_)_2_SO_4_ (50 μg N-NH_4_^+^ g^−1^ dry soil). Distilled water was added in each sample to achieve 24 ml of total liquid volume in flasks. The flasks were sealed with Parafilm^®^ and incubated at 28 °C under constant agitation (180 rpm). 1.5 ml of soil slurry were regularly sampled after 2, 4, 6, 8 and 10 h of incubation, filtered through 0.2 μm pore size and stored in vials at −20 °C until measurement of NO_3_^−^ concentrations using an ion chromatograph (DX120, Dionex, Salt Lake City, USA) equipped with a 4 × 250 mm column (IonPac AS9 HC). Based on the linear accumulation of NO_3_^−^ over time, NEA was expressed as μg N-NO_3_^−^ h^−1^ g^−1^ dry soil.

Soil ammonium concentration was determined by ion chromatography on soil extracts. They were prepared from a fresh soil subsamples equivalent of 5 g dry soil mixed with 20 ml of 0.1 M CaCl_2_ for 2 h at 10 °C under constant agitation (180 rpm) and filtered through 0.2 μm before analysis.

### Denitrification Enzymatic Activity (DEA)

DEA was measured according to Patra *et al.*^55^. Distilled water (1 ml) containing KNO_3_ (50 μg N-NO_3_^−^ g^−1^ dry soil), glucose (500 μg C-glucose g^−1^ dry soil) and glutamic acid (500 μg C-glutamic acid g^−1^ dry soil) was added to fresh soil (10 g equivalent dry soil) placed in a 150 ml plasma flask. The atmosphere was replaced by 90% helium to ensure anaerobic conditions and 10% C_2_H_2_ was added to inhibit N_2_O reductase activity. The flasks were sealed with rubber stoppers and incubated at 28 °C for 6 h. After two hours of incubation, gas samples were analyzed every hour for the remaining five hours to measure N_2_O concentration using a gas chromatograph (Micro GC R3000, SRA Instrument, Marcy L’Etoile, France). Based on the linear accumulation of N_2_O over time, DEA was expressed as μg N-N_2_O h^−1^ g^−1^ dry soil.

### DNA extraction and quantification

DNA was extracted from 0.5 g of flash frozen soil using the FastDNA™ SPIN Kit for Soil (MP Biomedicals laboratories) following the manufacturer’s instructions and then quantified using the Quant it™ Picogreen^©^ dsDNA Assay kit (Molecular Probes, USA). Fluorescence was measured using a UV spectrophotometer Xenius (Safas, Monaco) (λ = 520 nm). DNA extraction yields were not affected by TiO_2_-NP exposure (data not shown).

### Abundance of nitrifying bacteria (AOA and AOB)

The abundance of AOA and AOB was measured by quantitative PCR targeting *amoA* gene sequences encoding for an ammonia monooxygenase specific for each domain. Amplification was performed using gene primers amoA_1F and amoA_2R for the AOB^56^ and for the AOA^47^. The final reaction volume was 20 μl and final concentrations of reaction mix were: 0.5 μM of each primer for the bacterial *amoA* or 0.75 μM of CrenamoA616r and 1 μM of CrenamoA23f for the archaeal *amoA*, 2% bovine serum albumin (BSA), 1X of QuantiTect SybrGreen PCR Master Mix (Qiagen, Courtaboeuf, France) and 10 ng of soil DNA extract or of DNA standards with 10^2^ to 10^7^ gene copies μl^−1^ using a constructed linearized plasmid containing archaeal (54d9 fosmide fragment)^57^ and bacterial (*Nitrosomonas europaea*, GenBank accession number:L08050) *amoA* genes. The samples were run in duplicate on a Lightcycler 480 (Roche Diagnostics, Meylan, France) as follows: for bacterial *amoA* 15 min at 95 °C, 45 amplification cycles (30 s at 95 °C, 45 s at 54 °C, 45 s at 72 °C and 15 s at 80 °C) and 30 s at 40 °C; for archaeal *amoA*, 15 min at 95 °C, 50 amplification cycles (45 s at 94 °C, 45 s at 55 °C and 45 s at 72 °C) and 10 s at 40 °C. Melting curves analysis confirmed adequate amplification specificity.

### Denitrifying bacteria abundances

The abundance of denitrifying bacteria was measured by quantitative PCR of the genes encoding copper- and cytochrome cd1-containing nitrite reductase (*nirK* and *nirS*, respectively). Amplification was performed using nirK876 and nirK1040 gene primers^58^ or nirSCd3aF and nirSR3cd^59^. The final reaction volume for *nirK* quantification was 20 μL and final concentrations of reaction mix were: 1 μM of each primer, 0.02 μg of T4 gene protein 32 (QBiogene, Illkirch, France), 1X of QuantiTect SybrGreen PCR Master Mix (Qiagen, Courtaboeuf, France) and 5 ng of soil DNA extract or of standards with 10^2^ to 10^7^ copies of DNA (*Sinorhizobium meliloti* 1021). The samples were run in duplicate on a Lightcycler 480 (Roche Diagnostics, Meylan, France) as follows: 15 min at 95 °C, 45 amplification cycles (15 s at 95 °C, 30 s at 63 °C and 30 s at 72 °C) and 10 s at 40 °C. The final reaction volume for *nirS* quantification was 25 μl and final concentrations of reaction mix were: 0.5 μM of each primer, 0.02 μg of T4 gene protein 32 (QBiogene, Illkirch, France), 1X of QuantiTect SybrGreen PCR Master Mix (Qiagen, Courtaboeuf, France) and 12.5 ng of soil DNA extract or 10^2^–10^7^ copies of standard DNA (*Pseudomonas stutzeri* ATCC 14405). The samples were run in duplicate as follows: 15 min at 95 °C, 40 amplification cycles (15 s at 95 °C, 30 s at 66 °C, 30 s at 72 °C and 15 s at 80 °C) and 10 s at 40 °C. Melting curves analysis confirmed the amplification specificity.

### Diversity of bacteria, archaea and ammonia-oxidizers

High-throughput sequencing of bacterial and archaeal 16S rDNA genes and *amoA* AOB and AOA genes (Table 2) were performed on an Illumina MiSeq^®^ platform by Molecular Research DNA, USA. Sequencing was performed on the samples obtained after 7 or 90 d of incubation. A combination of the tools available from the RDP FunGene website^60^ and the open-source software Mothur (v.1.33.3)^61^ was used to process and analyze the sequence data (Table 2). Sequencing products were first paired in overlapping pair-ends, except for *amoA* (AOA) amplicons that were too long (>500 bp) for the MiSeq technology. Resulting sequence data were then sorted according to their length, and the quality of the primers (<2 errors) and barcodes (<1 error). The primers and barcodes were trimmed off before searching potential chimeric formation using UCHIME^62^ implemented in Mothur. Putative chimeras were removed from the dataset. For *amoA* sequences, nucleotide sequences were translated in amino-acids and possible frame-reading shifts were detected and corrected using the FRAMEBOT algorithm^63^.

In Mothur, we clustered the corrected 16S sequences into operational taxonomic units (OTUs) by setting a 0.03 distance limit^64^. The *amoA* sequences were clustered at 0.05 dissimilarity. The taxonomic identification of bacterial and archaeal 16S rDNA genes was performed using the Greengenes 13.5 database. Similarity of *amoA* sequences with known references were assessed with 7 652 sequences of *amoA* AOB and 9 326 sequences of *amoA* AOA extracted from the FunGene database^60^. The relatedness of *amoA* AOA sequences with identified archaeal taxa was performed with BLAST using the database assembled by Pester *et al.*^45^. Rarefaction curves based on identified OTU and species richness estimator ACE were generated using Mothur for each sample on a normalized number of sequences. Singleton reads were not considered for subsequent analyses. Non-metric Multi-Dimensional Scaling (NMDS) was performed using the *metaMDS* function, available in the Vegan package^65^ of the R software^66^ based on Bray-Curtis distances, associated to Permanova (Permutational multivariate analysis of variance) using the *adonis* function, to explore the difference in community composition in the different treatments.

### Statistical analysis

All results are presented as means (±standard errors). A one-way analysis of variance (ANOVA) and *post-hoc* Tukey HSD were performed to test the effect of TiO_2_-NP concentrations on measured variables at each sampling time. Where necessary, data were log-transformed prior to analysis to ensure conformity with the assumptions of normality and homogeneity of variances. Linear regressions were conducted to investigate relationships between all variables measured in the experiment, described by Spearman correlation coefficient (*r)*. Effects with *P* < 0.05 are referred to as significant. All statistical analyses were carried out using the R statistical software 2.13.2.

Path analysis^67^ was performed using Amos18^®^ (Amos Development Corporation, Crawfordville, FL, USA) to explore the causal links between TiO_2_-NP concentration, nitrifier and denitrifier abundances, nitrification and denitrification activities. A χ^2^ test was used to evaluate model fit, by determining whether the covariance structures implied by the model adequately fit the actual covariance structures of the data. A non-significant χ^2^ test (*P* > 0.05) indicates adequate model fits. The coefficients of each path as the calculated standardized coefficients were determined using the analysis of correlation matrices. These coefficients indicate by how many standard deviations the effect variable would change if the causal variable was changed by one standard deviation. The indirect effects of a variable can be calculated by the product of the coefficients along the path. Paths in this model were considered significant with a *P*-value < 0.05.

## Additional Information

**How to cite this article**: Simonin, M. *et al.* Titanium dioxide nanoparticles strongly impact soil microbial function by affecting archaeal nitrifiers. *Sci. Rep.* **6**, 33643; doi: 10.1038/srep33643 (2016).

## Supplementary Material

Supplementary Information

## Figures and Tables

**Table 1:** Correlation table between the different measured variables (AOB abundance, AOA abundance, *nirK* abundance, *nirS* abundance, NEA and DEA).

	AOB	AOA	*nirK*	*nirS*	NEA
AOB					
AOA	0.06 (0.69)				
*nirK*	0.04 (0.79)	−0.02 (0.94)			
*nirS*	0.22 (0.11)	**0.27 (0.03)**	−0.17 (0.20)		
NEA	−**0.43 (0.002)**	**0.51 (<0.001)**	**0.35 (0.02)**	0.01 (0.97)	
DEA	−**0.39 (0.004)**	0.20 (0.10)	**0.53 (<0.001)**	−0.08 (0.69)	**0.68 (<0.001)**

The strength of the linear relationship is given by the correlation coefficient *r*, *P*-values are given in parenthesis. Significant correlations are presented in bold. Spearman correlations were investigated on all data of the 3 sampling times (n = 54).

**Table 2:** Main information on the diversity analysis of bacterial, archaeal, AOB and AOA communities.

Community(Gene)	Primers	Mean Fragment size	Mean sequence number per sample	Total sequence number analyzed
Bacteria (16S rDNA)	515F/806R (V4 Region)	300 bp	4580 ± 166	160301
Archaea (16S rDNA)	349F/806R (V3-V4 Region)	424 bp	5745 ± 3332	206831
AOB (*amoA*)	amoA_1F/amoA_2R	435 bp	11745 ± 451	422844
AOA (*amoA*)	CrenamoA23F/CrenamoA616R	252 bp	16929 ± 9545	609456
