# Supplementary material for: Titanium dioxide nanoparticles strongly impact soil microbial function by affecting archaeal nitrifiers
Source: Sci Rep. 2016 Sep 23;6:33643. doi: 10.1038/srep33643 (PMC5034236; doi:10.1038/srep33643)
Supplement: Supplementary Information [file srep33643-s1.pdf]

SUPPLEMENTARY INFORMATION

Titanium dioxide nanoparticles strongly impact soil microbial function by affecting archaeal nitrifiers

Marie SIMONIN[1,2†], Agnès RICHAUME[1*], Julien GUYONNET[1], Audrey DUBOST[1], Jean M. F. MARTINS[2], and Thomas POMMIER[1]

[1] UMR CNRS 5557, Laboratoire d'Ecologie Microbienne, Université Lyon1, Université de Lyon, USC INRA 1364, bât G. Mendel, 43 boulevard du 11 novembre 1918, F-69622 Villeurbanne Cedex, France.

[2] LTHE (UMR CNRS 5564) - Université Joseph Fourier Grenoble 1, 38041 Grenoble Cedex 9, France

*For correspondence: agnes.richaume@univ-lyon1.fr.

†Present address: Department of Biology, Duke University, Durham, NC, USA; Center for the Environmental Implications of Nanotechnology, Duke University, Durham, NC, United States.

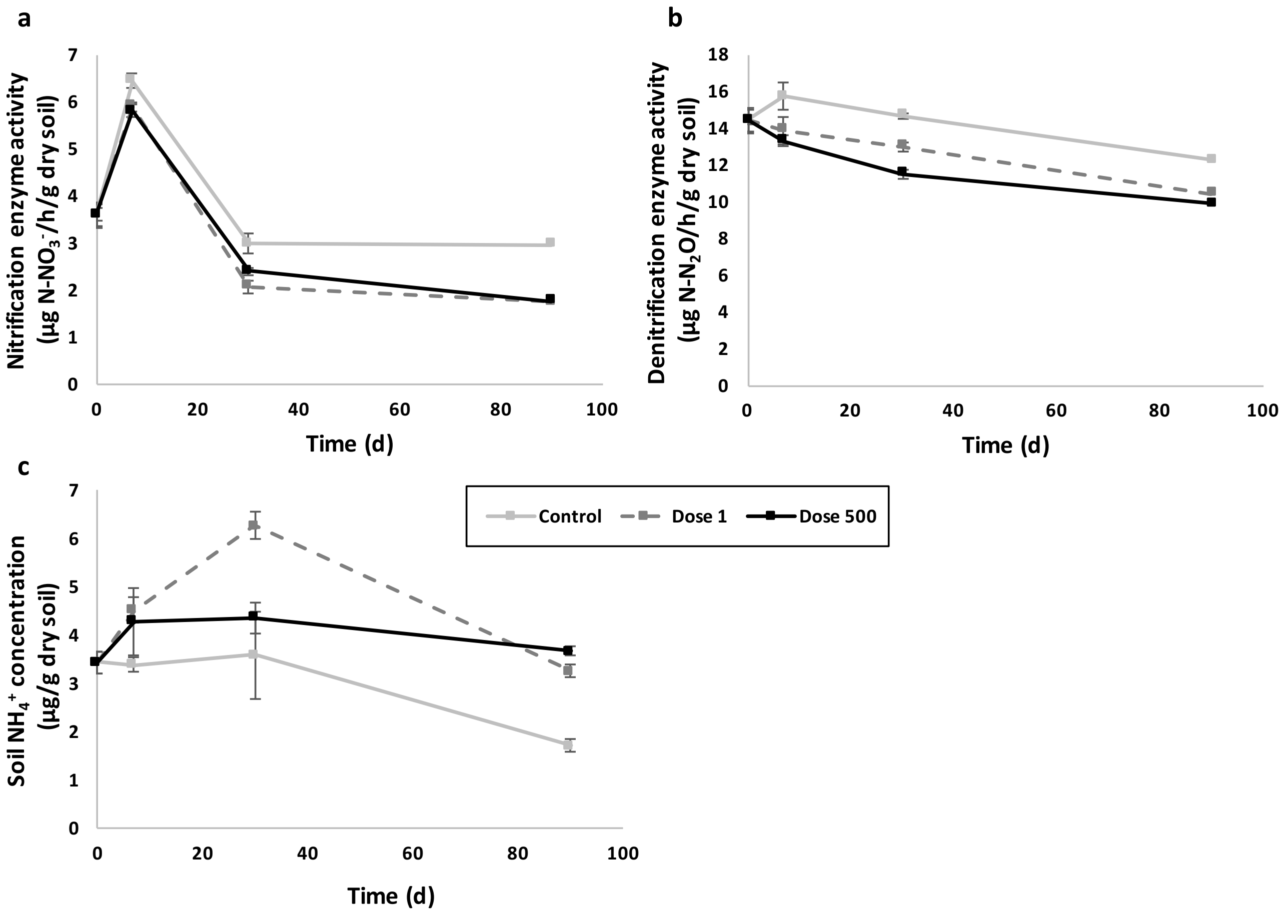

Figure S1: Dynamics over time of: a) NEA, b) DEA and c) soil NH_4^+ concentration in the control (grey line), Dose 1 mg kg^{-1} (dash line) and Dose 500 mg kg^{-1} (black line) of TiO_2 – NPs treatments.

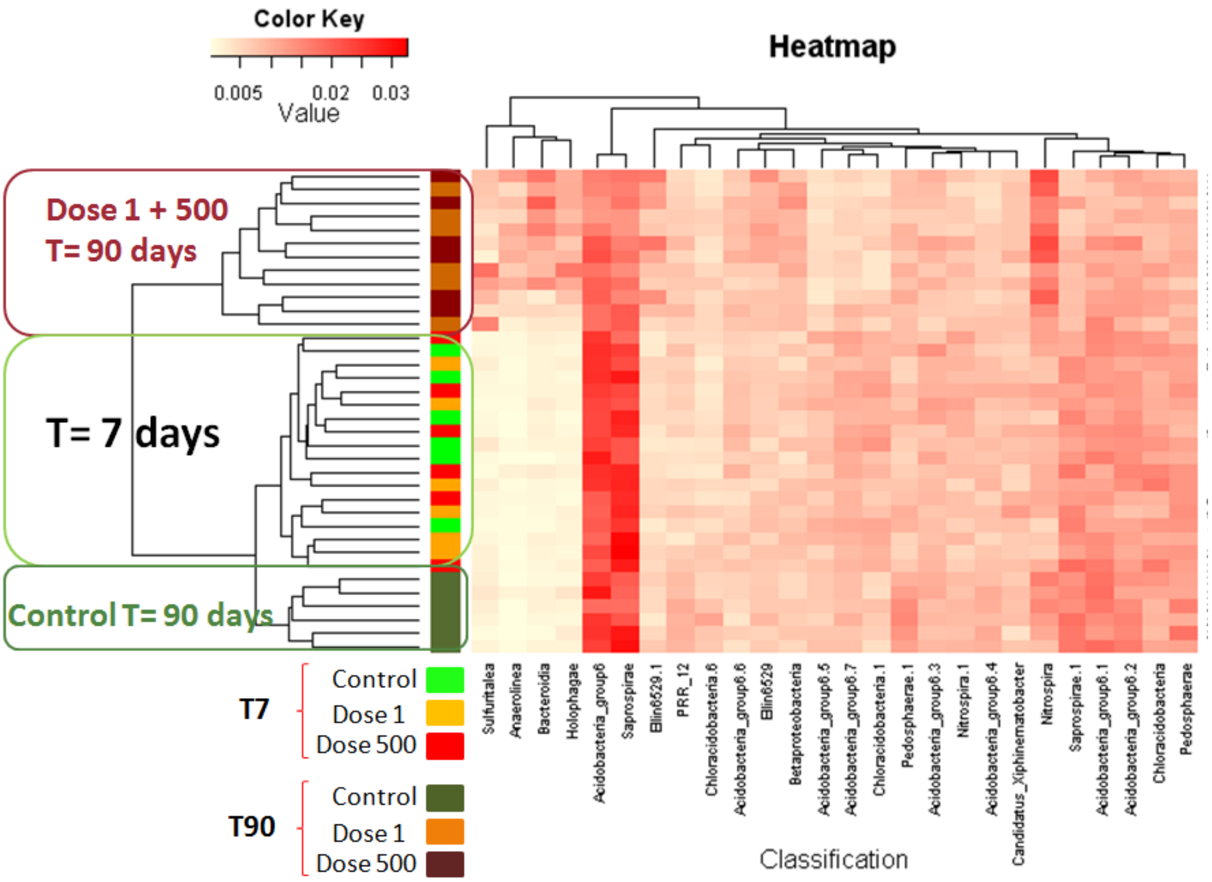

Figure S2 Relative abundance of bacterial OTUs in function of the different TiO_2-NPs concentrations and duration of incubation. The heatmap represents the proportions of OTUs at the genus level.

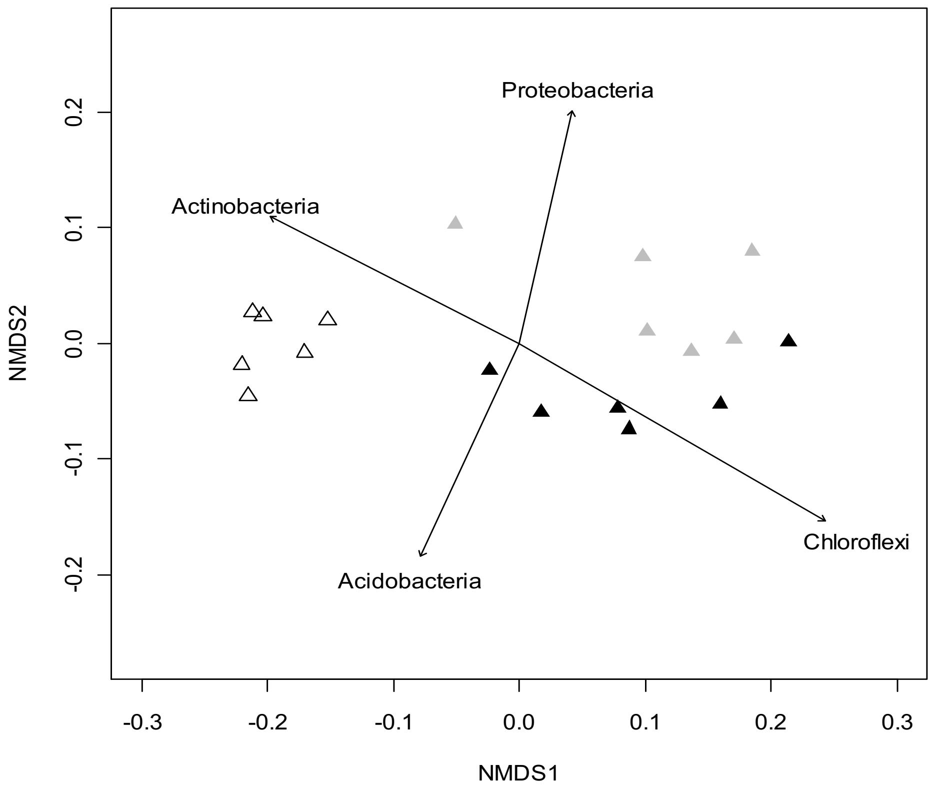

Figure S3 Nonmetric multidimensional scaling (NMDS) of the bacterial community structure after 90 days of exposure to TiO_2-NPs (control: white triangle; Dose 1: grey triangle; Dose 500: black triangle). Significant correlation between NMDS axis and bacterial phyla relative abundance, based on a post hoc permutation test (n = 999), are represented as arrows. The final stress for NMDS analysis was 0.06.

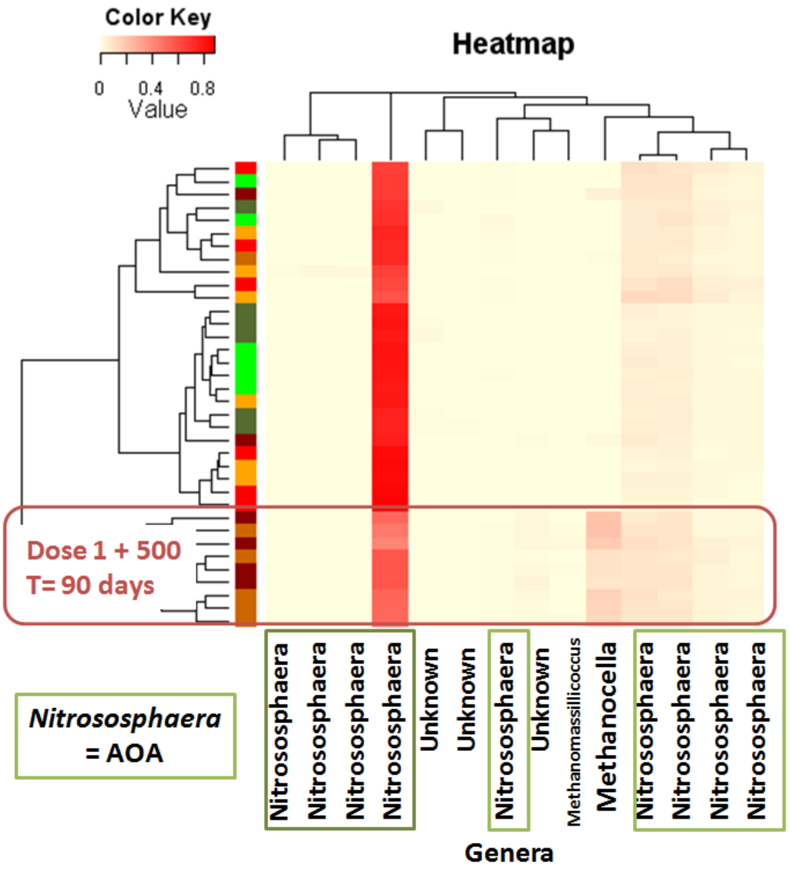

Figure S4 Relative abundance of archaeal genera in function of the different TiO_2-NPs concentrations and duration of incubation. The heatmap represents the proportions of OTUs at the genus level. See color legend on Figure S1.

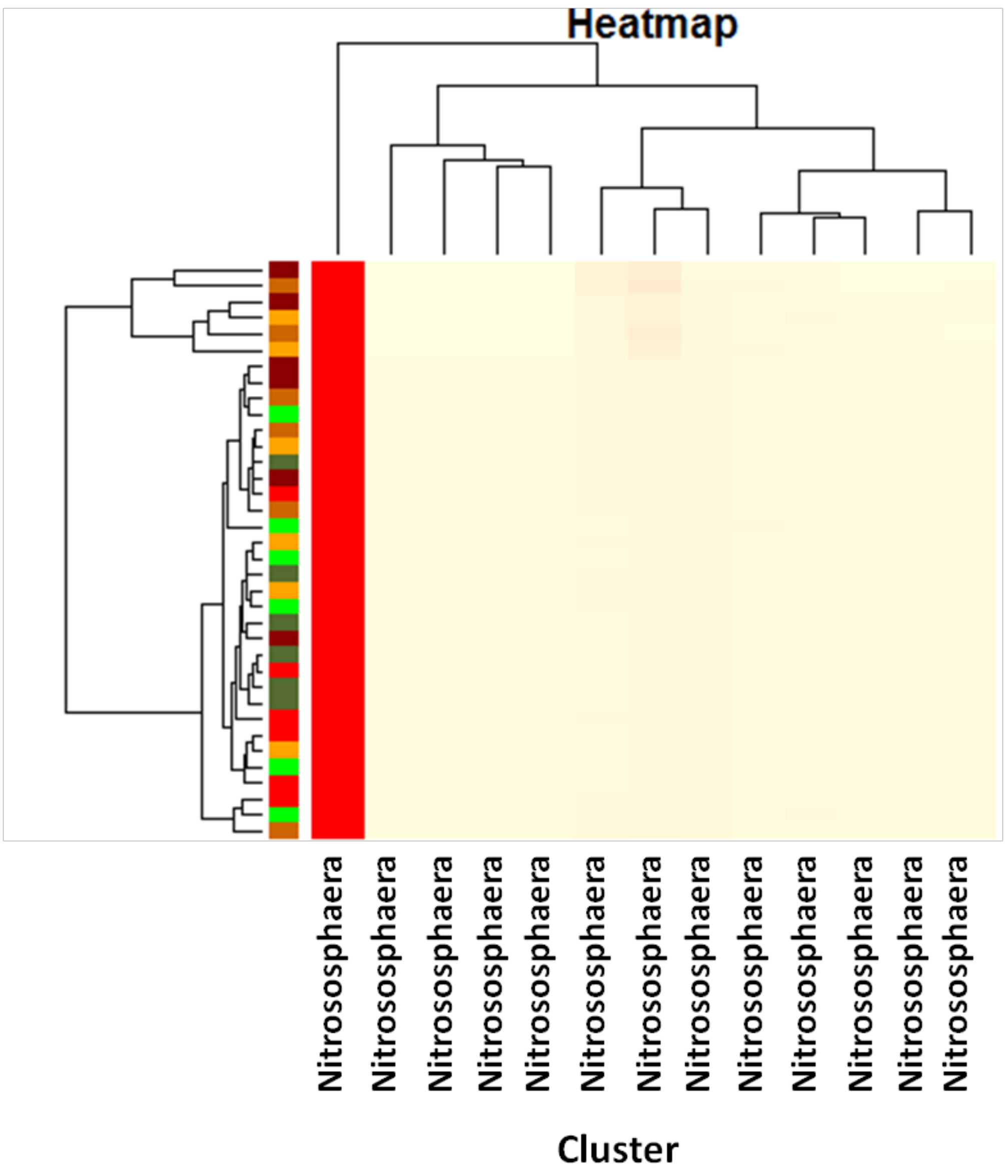

Figure S5 Relative abundance of AOA OTUs in function of the different TiO_2-NPs concentrations and duration of incubation. The heatmap represents the proportions of the different AOA clusters. See color legend on Figure S1.
